# A study on differences in the structure and function of the oral microbiome in 3- to 5-year-old children from plateau and plain regions’

**DOI:** 10.3389/froh.2026.1983860

**Published:** 2026-10-20

**Authors:** Xiaofan Bai, Lan Yang, Zhifei Zhou, Xiao Sun, Fen Liu, Ning Dong

**Affiliations:** 1 Key Laboratory of Shaanxi Province for Craniofacial Precision Medicine Research, College of Stomatology, Xi'an Jiaotong University, Xi’an, Shaanxi, China; 2 Clinical Research Center of Shaanxi Province for Dental and Maxillofacial Diseases, College of Stomatology, Xi’an Jiaotong University, Xi’an, Shaanxi, China; 3 Department of Stomatology, General Hospital of Tibetan Military Command, Lhasa, China; 4 Department of Pediatric Dentistry, College of Stomatology, Xi'an Jiaotong University, Xi’an, Shaanxi, China; 5 Department of Fourth Outpatient Department, College of Stomatology, Xi'an Jiaotong University, Xi’an, Shaanxi, China

**Keywords:** 16S rRNA gene sequencing, children;, early childhood caries, gingival inflammation, oral microbiota, plateau environment, saliva

## Abstract

**Background:**

The oral microbiome critically influences childhood caries and periodontal disease, yet how altitude and ethnicity shape it in preschool children remains unclear. This study aimed to investigate the associations of altitude and ethnicity on the composition, diversity, and metabolic pathways of the oral microbiome; analyze the potential associations between microbiome characteristics and dental caries and periodontal mucosal inflammation; and provide a microbiome-based theoretical foundation for the precise prevention and control of oral diseases in young children in high-altitude regions.

**Methods:**

This study included 87 children aged 3–5 years who were divided into three groups: low-altitude Han Chinese (*n* = 27), high-altitude Han Chinese (*n* = 30), and high-altitude Tibetan (*n* = 30). Demographic, dietary, oral hygiene, and family socioeconomic data were collected via questionnaires. Unstimulated saliva samples were collected for high-throughput 16S rRNA gene sequencing, and microbial community potential function was predicted using PICRUSt2 and the KEGG database. A multiple linear regression model was used to adjust for confounding variables and false discovery rate (FDR) correction was applied to assess the associations of altitude and ethnicity on oral microbial diversity, microbial abundance, and metabolic pathways.

**Results:**

High-altitude children had greater oral microbial alpha diversity, with all four indices remaining significant after FDR correction (all *q* < 0.01); ethnicity showed no independent effect. Altitude showed independent positive associations with *Bacteroidetes*, *Prevotella melaninogenica*, *Haemophilus parainfluenzae*, *Veillonella parvula*, and *Veillonella atypica* (*q* < 0.01); None of the six species-level associations with ethnicity remained significant after FDR correction (all *q* > 0.05). Functional prediction analysis revealed that the microbial communities of low-altitude children presented higher predicted enrichment of glycolysis and fatty acid metabolism pathways, whereas high-altitude children predicted enrichment of NOD-like receptor signaling and protein digestion and absorption pathways.

**Conclusion:**

Altitude shows strong statistical associations with oral microbial diversity and community structure in 3–5-year-old children, and the magnitude of these associations exceeded those for ethnicity-associated factors ethnicity-associated factors. Based on the association patterns between the microbiota and oral diseases, this study provides foundational data to support differentiated management and microbiome-based interventions for oral health in children living at different altitudes.

## 1 Background

The human microbiome is referred to as the “second human genome,” and its microbial homeostasis is closely related to host immunity, metabolism, and systemic health (1). As the second most complex symbiotic microbial community in the human body, an imbalance in the oral microbiome triggers the two most common oral diseases in children—dental caries and periodontal disease—and is regulated by multiple factors, including age, diet, oral hygiene, genetics, and living environment (2, 3). According to the Fourth National Oral Health Survey of China, the prevalence of caries in the primary dentition increased markedly with age, from 50.8% at 3 years to 63.6% at 4 years and 71.9% at 5 years; the corresponding mean dmft values were 2.28, 3.40, and 4.24, respectively (4). In contrast, destructive periodontitis is uncommon in preschool children. Gingival inflammation is more relevant to this developmental stage, although its reported prevalence varies considerably according to age, population, and diagnostic criteria (5). High-altitude regions are characterized by extreme environmental conditions such as low oxygen levels, low atmospheric pressure, low temperatures, and intense radiation (6). Long-term exposure to these conditions can reshape the human symbiotic microbial community, making these regions natural models for studying environment‒microbe interactions (7). However, evidence that residential altitude itself alters the prevalence of oral diseases in preschool children remains limited. A survey of 1,597 children aged 3–5 years in high-altitude Changdu reported an overall caries prevalence of 52.85% and a mean dmft of 2.44, with lower caries prevalence and dmft among 3-year-olds than among 4- and 5-year-olds (8).

Existing studies on the oral microbiome of plateau populations have focused primarily on adults and have already demonstrated altitude-associated variation in oral microbial composition. There are significant differences in the composition of the oral microbiome and the prevalence of periodontal disease between Han and Tibetan adults at different altitudes. Hao et al. reported altitude-related changes in specific bacterial taxa and suggested that environmental factors contribute to shaping oral microbial communities (9–12). Furthermore, studies have shown that the diversity and species richness of the salivary microbiome in Han Chinese people are greater than those in Tibetan people (10); however, since the adult microbiome has already reached a state of homeostasis, these findings cannot be extrapolated to the developmental patterns of the pediatric microbiome.

The establishment of the pediatric oral microbiome is a highly dynamic process: between ages 0 and 3, microbial composition is influenced primarily by feeding methods and dietary patterns; after age 3, environmental factors gradually become the main driving force, although microbial diversity in children at this stage generally remains lower than that in adults (13). Preschool children aged 3–5 years are in a critical window for the maturation of their oral microbiome, during which microbial colonization is extremely plastic. At this stage, environmental factors appear to be more strongly associated with microbiome profiles compared with genetic factors, and the composition of the microbiome can directly influence the risk of dental caries and periodontal and mucosal diseases in children. Overgrowth of *Streptococcus mutans* can lead to sustained acid production, inducing dental caries in young children, whereas enrichment of anaerobic pathogens such as *Prevotella, Porphyromonas*, and *Fusobacterium* may induce chronic gingival inflammation. An imbalance in either of these microbial communities can disrupt children's local mucosal immunity and even their systemic development (14). Currently, systematic studies on the oral microbiome of young children in high-altitude regions are scarce both domestically and internationally. The effects of altitude and ethnic genetic factors on children's oral microbiota remain unclear, and basic data on how these microbial differences translate to varying risks of dental caries and periodontal disease among children living at high and low altitudes are lacking.

To address these research gaps, this study examined healthy 3- to 5-year-old children from three groups: Han Chinese from low-altitude plains, Han Chinese from high-altitude regions, and Tibetan children from high-altitude regions. Using 16S rRNA high-throughput sequencing, we analyzed the composition and diversity characteristics of the salivary microbiome. Combined with functional prediction to identify differences in microbial metabolic pathways, we investigated the associations of altitude and ethnicity with the oral microbiome and explored potential links among “altitude environment–microbial community structure–susceptibility to childhood oral diseases,” thereby providing foundational baseline data for the prevention and control of oral diseases and targeted microbiome interventions in children of different altitudes and ethnicities.

## 2 Materials and methods

### 2.1 Ethical statement

This study adheres to the ethical guidelines of the Declaration of Helsinki and was approved by the Ethics Committee of the Stomatological Hospital of Xi' an Jiaotong University (2023-XJKQIEC-048-002). The legal guardians of all the participating children signed written informed consent forms. All experimental procedures were conducted in accordance with approved policies and regulations.

### 2.2 Study participants and grouping

#### 2.2.1 Sample source

We recruited Han children who had resided long term in the northwestern region (approximately 400 meters in altitude) since birth, as well as Han and Tibetan children who had resided long term in the plateau region (approximately 4,000 meters in altitude) since birth. They were divided into three groups: the low-altitude Han group (LH), the high-altitude Han group (HH), and the high-altitude Tibetan group (HT). As previous studies (10, 15, 16), 30 children were planned to be recruited for each group.

#### 2.2.2 Inclusion criteria

(1) Age 3–5 years; (2) no oral diseases such as missing teeth, carious teeth, pulpitis, or chronic periapical periodontitis; (3) no history of systemic diseases; (4) no use of systemic antibiotics, corticosteroids, or immunosuppressants within the past 3 months; (5) no use of topical oral antibiotics within the past week; and (6) informed consent from the child and their legal guardian.

#### 2.2.3 Exclusion criteria

(1) Acute oral inflammation or extensive mucosal lesions; (2) long-term use of probiotics or antibacterial mouthwash; (3) prolonged residence in another location over the past six months; or (4) inability of the guardian to complete the questionnaire and collect samples.

### 2.3 Questionnaire survey

Prior to participant enrollment, trained researchers explained the questionnaire content to the children's guardians, who then completed the questionnaire by scanning a QR code. The questionnaire covered baseline sociodemographic characteristics, dietary habits, oral hygiene behaviors, and health-related characteristics.

1. Sociodemographic characteristics: sex, place of residence, mother's educational attainment, mother's employment status, and family socioeconomic status (SES). SES was comprehensively assessed on the basis of the mother's educational attainment, employment status, and annual household income and was categorized into five levels (Levels 1–5) from highest to lowest scores (17).
2. Dietary indicators: Daily sugar intake was recorded continuously for three days, and the average was calculated; the method for calculating daily sugar intake from sugar-sweetened beverages was similar (18).
3. Oral hygiene behaviors: frequency of daily toothbrushing and whether fluoride toothpaste is used routinely.
4. Environmental exposure: urban or rural residence type and household tobacco exposure.

### 2.4 Saliva sample collection

All the participating children had informed consent forms signed by their legal guardians prior to sample collection and had not taken any antibiotics during this period. Eating, drinking, and any oral hygiene activities (including the use of mouthwash) were prohibited for at least 1 h prior to sampling. Prior to collection, participants gently rinsed their mouths with sterile water to remove food debris, after which 2 mL of saliva secreted under nonstimulated conditions was collected using a sterile centrifuge tube. After centrifugation, the saliva was aliquoted into 2 mL cryotubes and immediately transferred to a −80° C freezer for storage in preparation for subsequent testing.

### 2.5 16S rRNA V3–V4 region amplification and illumina novaseq sequencing

DNA samples were extracted using a modified cetyl-trimethyl-ammonium bromide (CTAB) method. The V3–V4 region of the bacterial 16S rRNA gene, which is highly variable, was selected as the sequencing target. This region has been widely used in community classification and identification studies of human microbiome-related samples (19). For PCR amplification, we used primers specific to the bacterial 16S rRNA V3–V4 region: 338F (5′-barcode + ACTCCTACGGGAGGCAGCA-3′) and 806R (5′-GACTACHVGGGTWTCTAAT-3′). The purified amplification products were mixed in equimolar proportions. Raw sequencing data were quality-assessed using FastQC software, followed by sequencing on the Illumina NovaSeq platform (Illumina, San Diego, CA, USA) using the NovaSeq 6000 SP Reagent Kit (500 cycles) to analyze the composition of the oral microbiome. To ensure sequencing quality, the optimal sequencing length for target fragments was set at 200–450 bp.

### 2.6 Prediction of microbial metabolic functions

The PICRUSt2 tool was used to predict the potential metabolic functions of the microbial community on the basis of the KEGG database (http://www.kegg.jp/). On the basis of the predicted functional unit abundance matrix, differences in functional composition between groups were compared using principal coordinate analysis (PCoA), and differential metabolic pathways were identified through enrichment analysis. Metabolic pathways showing significant differences between groups were determined on the basis of the adjusted *P* value (adj. P) and the log₂ fold change (log_2_FC), while the species contribution of the microbial community at the genus level within each metabolic pathway was also analyzed.

### 2.7 Sequencing data processing

Microbiome biological information was analyzed using QIIME2 (version 2024.5), following a modified and optimized workflow based on the official tutorial (https://docs.qiime2.org/). Raw sequence data were demultiplexed using the demuxer plugin followed by primers cutting with cut adapt plugin. Sequences were then quality filtered, denoised, merged and chimera removed using the DADA2 plugin. Sequences outside the 200–450 bp length range were discarded. On average, each sample yielded 85547 valid reads, with 1.1 % of raw reads removed during filtering. All samples were rarefied to a depth of 45,620 reads. By utilizing the Greengenes database, the amplicon sequence variant (ASV) feature sequences were aligned against the reference sequences in the database to obtain taxonomic information corresponding to each ASV. The abundance matrix with rare ASVs removed was used for a series of subsequent analyses.

QIIME2 was employed to calculate the alpha diversity indices for each sample individually. For beta diversity analysis, R software and QIIME2 were used, with the UniFrac distance metric applied to investigate variations in microbial community structure among samples. This analysis was visualized via principal coordinate analysis (PCoA). Moreover, principal component analysis (PCA) was conducted based on the species composition profile at the genus level. PERMANOVA (Adonis/PERMANOVA) was used to assess the significance of differences in microbial community structure among groups.

### 2.8 Statistical analysis

All the statistical analyses were performed using SPSS 29.0 software. *A P value* of <0.05 was considered to indicate statistical significance, and all tests were two-tailed. To account for multiple testing across related outcomes, raw *P* values were adjusted using the Benjamini-Hochberg false discovery rate (FDR) procedure.

#### 2.8.1 Analysis of baseline characteristics

The continuous variable (age) did not follow a normal distribution and is expressed as the median (interquartile range); the Kruskal–Wallis *H*-test was used for comparisons between groups. Categorical variables are expressed as frequencies and percentages (%); the Pearson chi-square test was used for comparisons between groups, and Fisher's exact test was used when the expected frequency was less than 5.

#### 2.8.2 Multivariate linear regression analysis

The LH group was designated the “low-altitude group,” and the LH and HH groups were combined into the “Han Chinese group.” Using the low-altitude group and the Han Chinese group as reference groups, two independent variables—“altitude” (low altitude = 0, high altitude = 1) and “ethnicity” (Han Chinese = 0, Tibetan = 1)—were established. After adjusting for baseline confounding factors, the independent effects of altitude and ethnicity on microbiome indices were evaluated.

## 3 Results

### 3.1 Comparison of baseline characteristics (comparison of baseline characteristics of the included participants)

As shown in Table 1, this study included a total of 87 eligible children, who were divided into a low-altitude Han Chinese group (LH, *n* = 27), a high-altitude Han Chinese group (HH, *n* = 30), and a high-altitude Tibetan group (HT, *n* = 30). There were no statistically significant differences among the three groups in terms of median age, sex distribution, daily toothbrushing frequency, use of fluoride toothpaste, household socioeconomic status, or tobacco exposure (*P* > 0.05). There were significant differences in the frequency of sugar intake among the different groups (*P* < 0.05), with the LH group having the highest proportion of high-frequency consumers (37.04%). In addition, dietary pattern, residential area, maternal education level, and employment status were significantly different across the groups (*P* < 0.05).

**Table 1:** Comparison of baseline characteristics.

Characteristic	Total (*n* = 87)	LH (*n* = 27)	HH (*n* = 30)	HT (*N* = 30)	*Χ*^2^/H	*P* value^2^
Sex, *n* (%)^a^					^b^0.07	0.97
Male	48 (55.17)	15 (55.56)	17 (56.67)	16 (53.33)		
Female	39 (44.83)	12 (44.44)	13 (43.33)	14 (46.67)		
Age [years, Median (Q1, Q3)]^c^	4.00 (3.00, 5.00)	4.00 (3.00, 5.00)	4.00 (4.00, 5.00)	4.00 (3.00, 5.00)	^d^0.66	0.72
Number of times teeth are brushed per day, *n* (%)^a^					/	0.253
More than 2 times	1 (1.15)	1 (3.70)	0 (0.00)	0 (0.00)		
2 times	29 (33.33)	12 (44.44)	9 (30.00)	8 (26.67)		
1 time	57 (65.52)	14 (51.85)	21 (70.00)	22 (73.33)		
Occasionally or never	0 (0.00)	0 (0.00)	0 (0.00)	0 (0.00)		
Tobacco exposure in the family environment, *n* (%)^a^					^b^0.80	0.67
No	65 (74.71)	20 (74.07)	21 (70.00)	24 (80.00)		
Yes	22 (25.29)	7 (25.93)	9 (30.00)	6 (20.00)		
Daily sugar intake (g), *n* (%)^a^					/	<0.01
50–100	16 (18.39)	10 (37.04)	4 (13.33)	2 (6.67)		
100–150	40 (45.98)	15 (55.56)	11 (36.67)	14 (46.67)		
150–200	31 (35.63)	2 (7.41)	15 (50.00)	14 (46.67)		
Daily sugar intake from sweetened beverages (g), *n* (%)^a^					/	0.76
10–20	37 (42.53)	10 (37.04)	13 (43.33)	14 (46.67)		
20–30	37 (42.53)	11 (40.74)	14 (46.67)	12 (40.00)		
> 30	13 (14.94)	6 (22.22)	3 (10.00)	4 (13.33)		
Fluoride use, *n* (%)^a^	^b^2.31	0.32				
Regular	38 (43.68)	9 (33.33)	16 (53.33)	13 (43.33)		
None or irregular	49 (56.32)	18 (66.67)	14 (46.67)	17 (56.67)		
Dietary pattern, *n* (%)^a^					^b^10.64	0.03
Animal based	44 (50.57)	8 (29.63)	17 (56.67)	19 (63.33)		
Animal–plant balanced	26 (29.89)	9 (33.33)	8 (26.67)	9 (30.00)		
Plant based	17 (19.54)	10 (37.04)	5 (16.67)	2 (6.67)		
Residential area, *n* (%)^a^					^b^9.58	0.01
City	43 (49.43)	20 (74.07)	12 (40.00)	11 (36.67)		
Rural	44 (50.57)	7 (25.93)	18 (60.00)	19 (63.33)		
Maternal education level, *n* (%)^a^					/	0.00
High school education or less	31 (35.63)	5 (18.52)	15 (50.00)	11 (36.67)		
Undergraduate education	44 (50.57)	12 (44.44)	14 (46.67)	18 (60.00)		
Graduate education and above	12 (13.79)	10 (37.04)	1 (3.33)	1 (3.33)		
Maternal employment status, *n* (%)^a^					^b^7.06	0.03
Employed	46 (52.87)	20 (74.07)	13 (43.33)	13 (43.33)		
Unemployed	41 (47.13)	7 (25.93)	17 (56.67)	17 (56.67)		
Family SES level^a^					/	0.94
1	12 (13.79)	4 (14.81)	4 (13.33)	4 (13.33)		
2	18 (20.69)	7 (25.93)	5 (16.67)	6 (20.00)		
3	32 (36.78)	8 (29.63)	13 (43.33)	11 (36.67)		
4	13 (14.94)	4 (14.81)	4 (13.33)	5 (16.67)		
5	12 (13.79)	4 (14.81)	4 (13.33)	4 (13.33)		

When the expected frequency was less than 5, Fisher's exact test was used. Significance notation: *P < 0.05.

a Presented as the median (M) and interquartile range (IQR).

b *χ*^2^ value (chi-square statistic).

c Presented as the frequency (n) and percentage (%).

d H value (Kruskal–Wallis *H*-test).

LH, Han group at low altitude; HH, Han group at high altitude; HT, Tibetan group at high altitude; SES, Socioeconomic status.

### 3.2 Characteristics of oral microbial community species composition

The results of 16S rRNA sequencing (Figures 1A,B) revealed that at the phylum level, the oral microbiota of all three groups was dominated by Firmicutes, Bacteroidetes, Proteobacteria, Actinobacteria, and Fusobacteria. Children in the HH and HT groups had significantly greater abundances of the Bacteroidetes and Fusobacteria phyla than children in the LH group did. At the genus level (Figure 1C), the major genera included *Streptococcus, Prevotella, Neisseria, Veillonella, Haemophilus, Gemella, Rothia, Porphyromonas, Fusobacterium*, and *Leptotrichia (Leptotrichia).* In the LH group, *Streptococcus, Gemella,* and *Rothia* were the predominant genera, whereas in the HH and HT groups, *Prevotella* was the dominant genus, followed by *Veillonella* and *Fusobacterium*.

**Figure 1:**
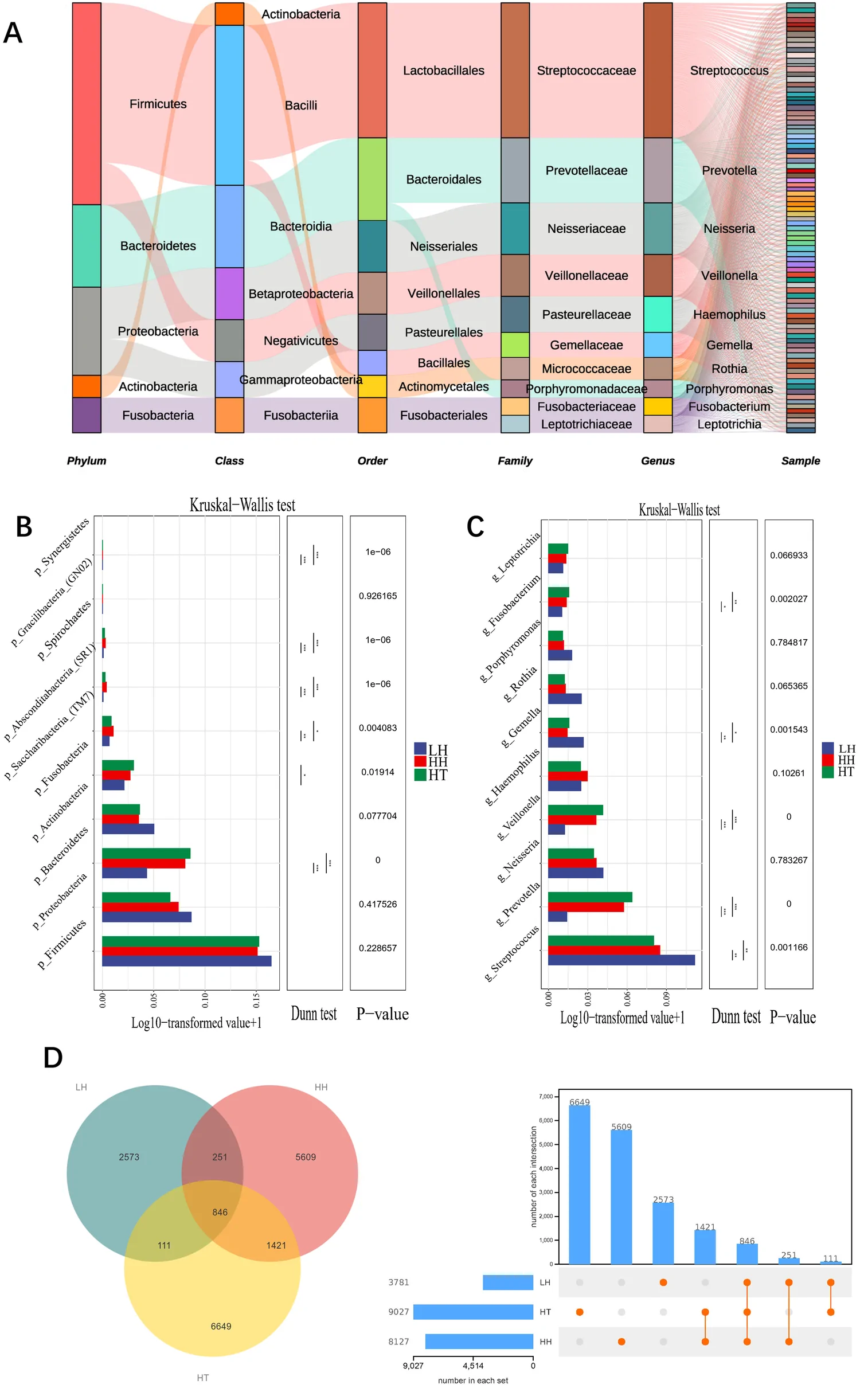
Analysis of the species composition and taxonomic composition of the oral microbiome: **(A)** species composition scatter plot; **(B)** differences in salivary microbial composition at the phylum level among the LH, HH, and HT groups; **(C)** differences in salivary microbial composition at the genus level among the LH, HH, and HT groups; **(D)** taxonomic composition analysis; venn diagram and upSet plot of species composition.

The ASV Venn diagram (Figure 1D) illustrates the shared and unique ASVs across the three groups (LH, HT, HH). Unique ASVs are those detected exclusively within one group, whereas overlapping regions represent ASVs shared by two or three groups. A total of 3,781 ASVs were detected in the LH group, 9,027 in the HT group, and 8,127 in the HH group. Among them, 2,573 ASVs were unique to LH, 6,649 unique to HT, and 5,609 unique to HH. There were 251 ASVs shared only by LH and HH, 1,421 ASVs shared only by HH and HT, and 111 ASVs shared only by LH and HT. A total of 846 core ASVs were present in all three groups, accounting for 9.37%–22.38% of the ASVs within each respective group. Children residing at high altitudes harbored more ASVs than those in the low-altitude LH group, with the HT group showing the highest ASV richness.

### 3.3 Analysis of α- and β-diversity

#### 3.3.1 α-diversity analysis

The Chao-1 and Observed Species indices were used to assess microbial richness, while the Shannon and Simpson indices were used to assess diversity. As shown in Figures 2A,B, the Chao-1, Observed Species, Shannon, and Simpson indices in the LH group were significantly lower than those in the HH and HT groups (*P* < 0.05), whereas no significant differences were observed between the HH and HT groups.

**Figure 2:**
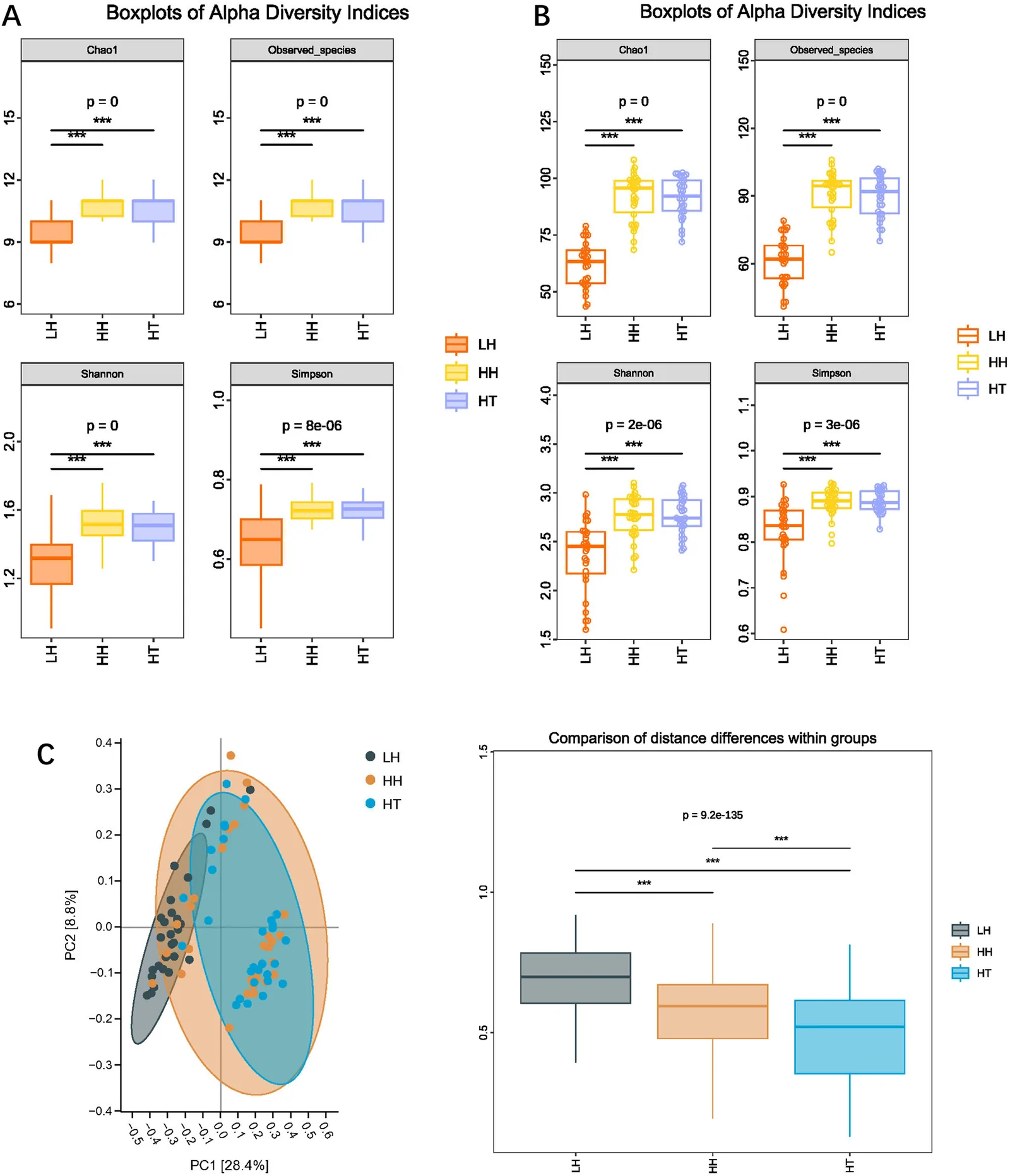
Diversity analysis **(A)** phylogenetic level: alpha diversity index for the LH, HH, and HT groups; **(B)** genetic level: alpha diversity index for the LH, HH, and HT groups; **(C)** PCoA. Data are expressed as the mean ± standard deviation.

As shown in Table 2, after adjusting for all baseline confounders via multiple linear regression, altitude was positively associated with all four alpha-diversity indices, and all four associations remained significant after Benjamini-Hochberg FDR correction (B = 0.74–0.76, *P* < 0.01); the ethnicity variable had no independent statistical effect on any of the α-diversity indices (*P* > 0.05).

**Table 2:** Results of multivariate linear regression for α-diversity.

Independent variables	Chao1 B (*P* value)	Observed_species B (*P* value)	Shannon B (*P* value)	Simpson B (*P* value)
Constant	— (0.02)	— (0.01)	— (<0.01)	— (<0.01)
Daily sugar intake	0.04 (0.65)	0.05 (0.56)	0.05 (0.56)	−0.06 (0.53)
Dietary pattern	0.00 (0.96)	0.00 (0.97)	−0.06 (0.49)	0.09 (0.33)
Residential area	−0.04 (0.66)	−0.05 (0.53)	0.00 (0.96)	−0.04 (0.65)
Maternal Education level	0.02 (0.84)	0.01 (0.88)	0.02 (0.84)	0.10 (0.31)
Maternal employment status	−0.00 (0.99)	−0.01 (0.90)	−0.05 (0.48)	−0.02 (0.86)
Elevation	0.75 (<0.01)	0.74 (<0.01)	0.75 (<0.01)	0.76 (<0.01)
Ethnicity	0.04 (0.67)	0.05 (0.59)	0.01 (0.89)	0.04 (0.67)

The four altitude-effect *P* values were adjusted as one family using the Benjamini-Hochberg procedure; all remained significant (all q < 0.01).

B, Standardized regression coefficient beta.

#### 3.3.2 β-diversity analysis

As shown in Figure 2C, PCoA based on the Bray‒Curtis distance revealed that sample communities formed distinct clusters according to elevation, with PC1 explaining 28.4% of the community variation and PC2 explaining 8.8%; the Han Chinese community at low altitudes was most distinctly separated from the two high-altitude sample groups, while the HH and HT samples showed some overlap, and intergroup differences still existed. PERMANOVA confirmed that the overall structure of the microbial communities differed significantly across the three groups (*P* < 0.05) and that altitude was the primary factor driving community differentiation.

### 3.4 Analysis of characteristic microbial communities in the oral microbiome

#### 3.4.1 Differences in the microbial communities between the LH and HH groups

LEfSe analysis and LDA scores jointly revealed differential microbial communities between the LH and HH groups (Figure 3A). The LH group was enriched in the phylum Actinobacteria, order *Lactobacillales*, genus *Streptococcus*, and genus *Gemella*, whereas the HH group was enriched in the phylum *Bacteroidetes*, the class *Negativicutes*, the phylum *Saccharibacteria*, the family *Veillonellaceae*, and the species *Prevotella melaninogenica*.

**Figure 3:**
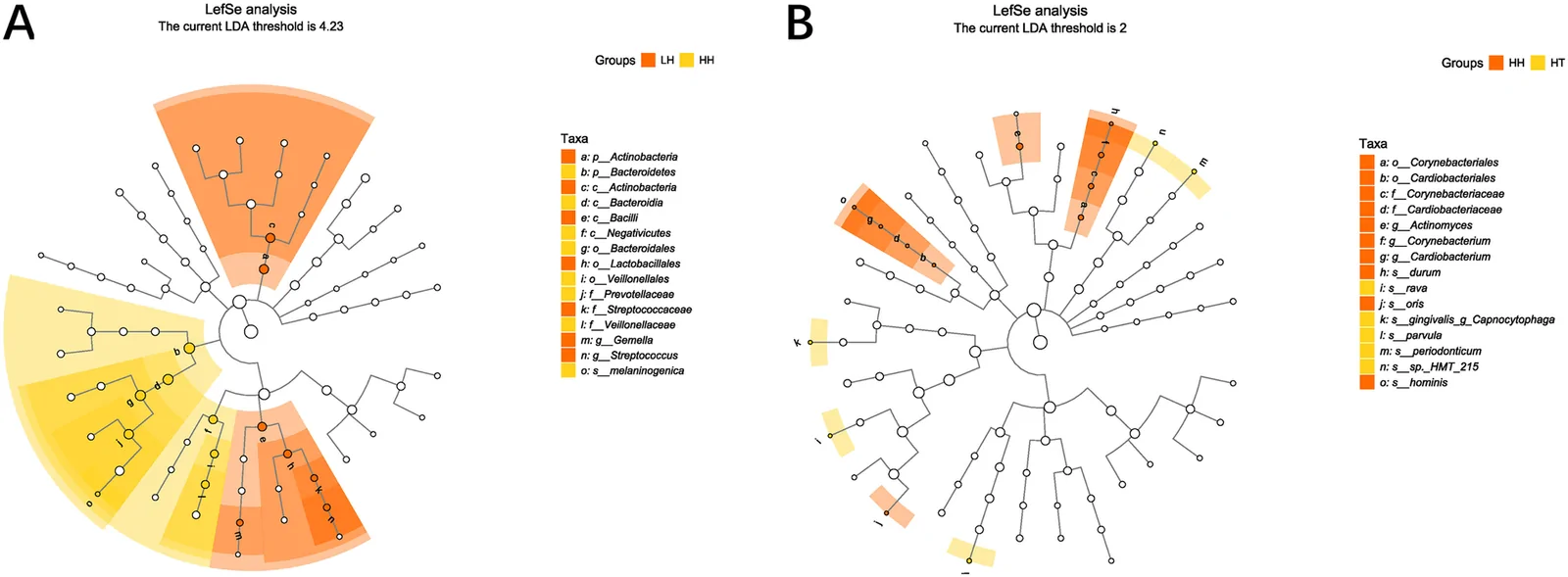
Characteristic bacterial communities in saliva across different groups **(A)** LH and HH. **(B)** HH and HT.

#### 3.4.2 Differences in microbial communities between the HH and HT groups

The differential microbial communities between the HH and HT groups are shown in Figure 3B. The enrichment of *Porphyromonas gingivalis*, *Fusobacterium periodonticum*, and Capnocytophaga was detected in the HT group, whereas Actinomyces, Corynebacterium, and Cardiobacterium were enriched in the HH group.

#### 3.4.3 Independent effects of altitude and ethnicity on dominant microbial communities

To analyze the independent contributions of altitude and ethnicity to microbial community composition, the LH and HH groups were combined into the “Han Chinese group.” Using low altitude and Han Chinese as the reference, two independent variables—“altitude” (0 = low altitude, 1 = high altitude) and “ethnicity” (0 = Han Chinese, 1 = Tibetan)—were constructed, and the analysis was performed after all baseline confounders were adjusted.

At the phylum level (Table 3), altitude was positively associated with Bacteroidetes, and this association remained significant after FDR correction (B = 0.80, *P* < 0.001, *q* < 0.001). The negative association between altitude and Firmicutes was nominally significant before correction (B = −0.35, *P* = 0.028) but did not remain significant after FDR correction (*q* = 0.056); no significant association was observed with the Proteobacteria phylum. Ethnicity, however, had no significant independent effect on any of the dominant phyla at the phylum level (all *P* > 0.05).

**Table 3:** Results of multivariate linear regression at the phylum level.

Independent variables	p_Firmicutes B (*P*)	p_Proteobacteria B (*P*)	p_Bacteroidetes B (*P*)	p_Actinobacteria B (*P*)
Constant	— (<0.01)	— (<0.01)	— (0.06)	— (0.36)
Daily sugar intake	0.14 (0.27)	−0.11 (0.38)	−0.05 (0.62)	−0.02 (0.89)
Dietary pattern	−0.08 (0.48)	−0.19 (0.09)	0.25 (0.01)	0.28 (0.01)
Residential area	0.17 (0.16)	−0.20 (0.09)	−0.15 (0.09)	0.09 (0.42)
Maternal education level	0.05 (0.71)	−0.13 (0.26)	0.10 (0.27)	0.09 (0.41)
Maternal employment status	−0.03 (0.82)	0.02 (0.88)	−0.07 (0.39)	0.14 (0.20)
Elevation	−0.35 (0.03)	−0.20 (0.20)	0.80 (<0.01)	−0.27 (0.07)
Ethnicity	0.00 (0.98)	−0.10 (0.42)	0.11 (0.23)	0.04 (0.74)

*P* values are unadjusted and are presented for transparency. Bold values indicate associations that remained significant after Benjamini–Hochberg FDR correction (*q* < 0.05). Exact *q* values are reported in the Results section.

B, Standardized regression coefficient beta.

The regression results at the species level (Table 4) further revealed that altitude had a significant positive effect on the prevalence of *Prevotella melaninogenica* (B = 0.*66*, *P* < 0.01, *q* < 0.01), *Haemophilus parainfluenzae* (B = 0.*60*, *P* < 0.01, *q* < 0.01), *Veillonella parvula* (B = 0.*39*, *P* < 0.01, *q* < 0.01), and *Veillonella atypica* (B = 0.*58*, *P* < 0.01, *q* < 0.01); it had a significant negative effect on *Granulicatella adiacens* (B = −0.31, *P* = 0.02, *q* = 0.03) after FDR correction. The association with *Fusobacterium periodonticum* did not reach statistical significance (B = 0.27, *P* = 0.06, *q* = 0.06). None of the six species-level associations with ethnicity remained significant after FDR correction (*q* values = 0.08, 0.10, 0.24, 0.32, 0.66, and 0.88).

**Table 4:** Results of multivariate linear regression at the species level.

Independent variables	s_melaninogenica B (*P*)	s_parainfluenzae B (*P*)	s_parvula B (*P*)	s_atypica B (*P*)	s_adiacens B (*P*)	s_periodonticum B (*P*)
Constant	– (0.54)	– (0.06)	– (0.69)	– (0.81)	– (0.02)	– (0.45)
Daily sugar intake	−0.09 (0.39)	−0.14 (0.23)	−0.05 (0.64)	−0.08 (0.48)	−0.13 (0.21)	−0.06 (0.60)
Dietary pattern	0.25 (0.01)	0.19 (0.09)	−0.07 (0.47)	0.05 (0.64)	0.15 (0.13)	−0.07 (0.51)
Residential area	−0.16 (0.11)	−0.24 (0.04)	0.15 (0.13)	0.04 (0.73)	−0.09 (0.37)	0.18 (0.12)
Maternal education level	0.14 (0.16)	0.14 (0.22)	−0.00 (0.97)	0.10 (0.40)	0.21 (0.05)	0.02 (0.86)
Maternal employment status	0.06 (0.51)	−0.10 (0.36)	0.06 (0.51)	0.06 (0.56)	0.05 (0.58)	0.03 (0.75)
Elevation	0.66 (<0.01)	0.60 (<0.01)	0.39 (<0.01)	0.58 (<0.01)	−0.31 (0.02)	0.27 (0.06)
Ethnicity	0.25 (0.01)	−0.15 (0.22)	0.22 (0.03)	0.07 (0.55)	−0.02 (0.88)	0.18 (0.12)

*P* values are unadjusted and are presented for transparency. Bold values indicate associations that remained significant after Benjamini–Hochberg FDR correction (*q* < 0.05). Exact *q* values are reported in the Results section.

B, Standardized regression coefficient beta.

### 3.5 Potential links between microbial classification and functional pathways

Functional prediction was performed using PICRUSt2 on the basis of the KEGG database (Figure 4). Among the first-level functional pathways, significant differences were observed between the LH and HH groups, as well as between the HH and HT groups, in the categories of metabolism, cellular processes, genetic information processing, environmental information processing, human diseases, and organ systems (Figure 4A). Among the second-level functional pathways, cofactor and vitamin metabolism, carbohydrate metabolism, and amino acid metabolism were the most abundant (Figure 4B).

**Figure 4:**
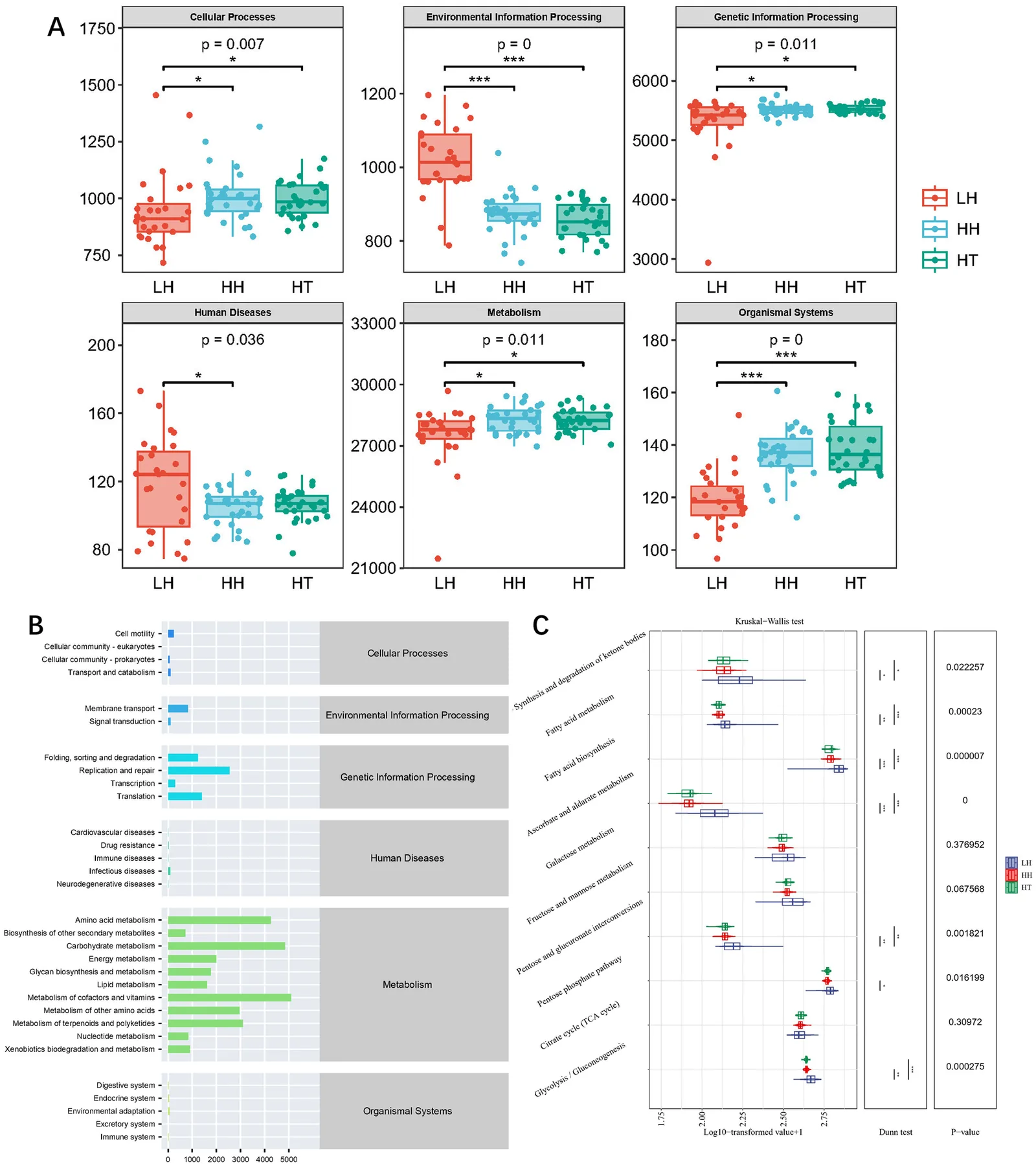
Functional prediction analysis of the oral salivary microbiota. **(A)** Comparison of PICRUSt2-predicted KEGG functional data; **(B)** metabolic histogram; **(C)** PICRUSt2-based functional prediction analysis of salivary microorganisms.

Analysis of metabolite pathway differences between groups (Figure 4C) revealed that compared with the high-altitude group, the LH group exhibited higher predicted enrichment of pathways such as glycolysis/gluconeogenesis, pentose-glucuronic acid interconversion, ascorbic acid and aldonic acid metabolism, fatty acid biosynthesis, and fatty acid metabolism. Conversely, the NOD-like receptor signaling pathway and protein digestion and absorption pathways were predicted to be more enriched in the HH and HT groups.

Analysis of the contribution of genera (Figure 5) indicated that the genera *Streptococcus, Prevotella melaninogenica*, Gemella, *Streptococcus salivarius*, and *Fusobacterium periodonticum* were the major contributing groups to the differentially expressed functional pathways.

**Figure 5:**
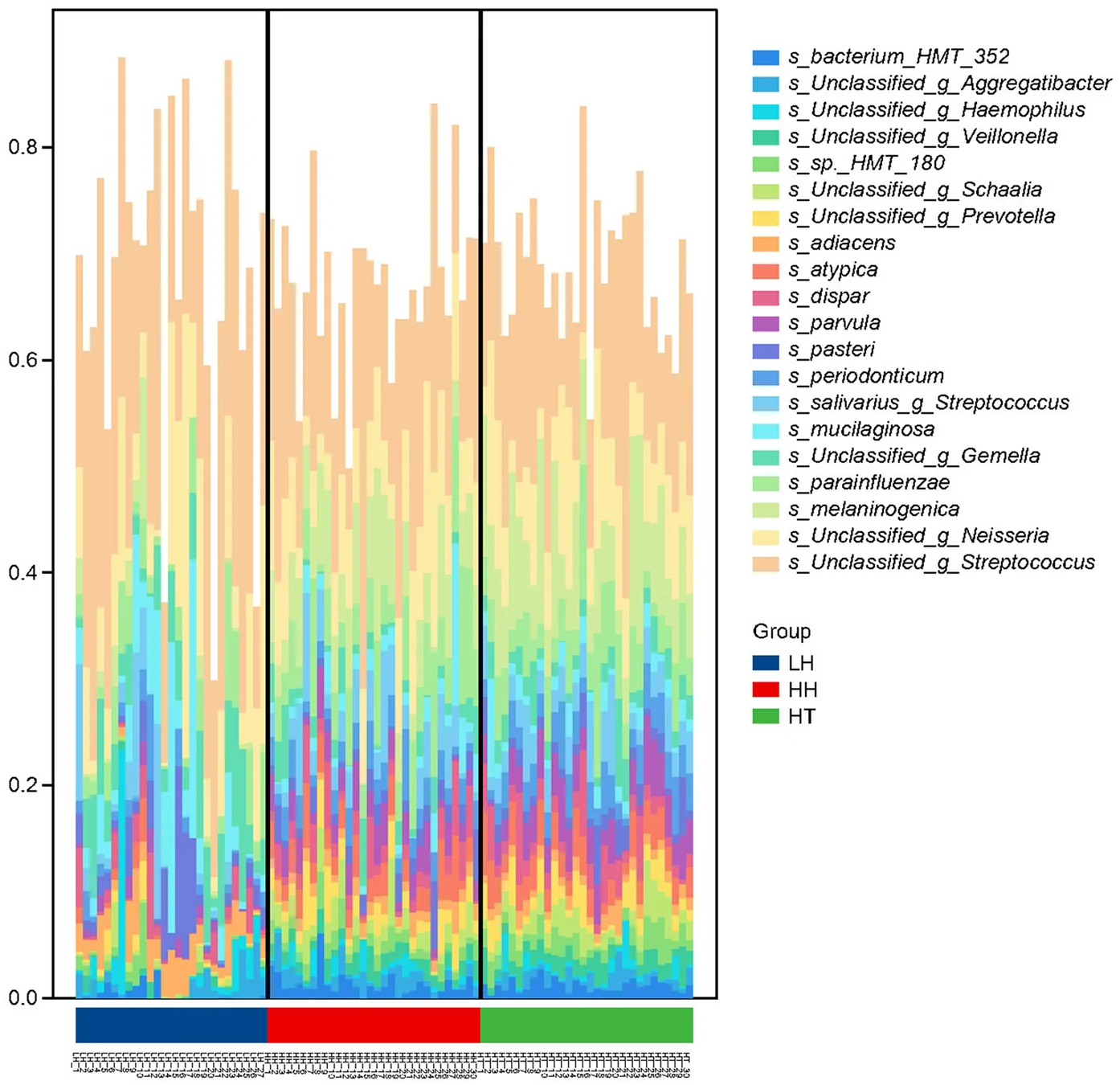
Contribution of genera within the genus to metabolic pathways. The x-axis represents sample labels, with different colors indicating their group affiliations; the y-axis represents the relative abundance of the relevant metabolic pathway; and the contribution ratios of different taxonomic units within the genus to this metabolic pathway are displayed in distinct color layers.

## 4 Discussion

As an integral component of the human symbiotic system, microorganisms profoundly influence host health and disease states (20). The homeostasis of the oral microbiome serves as a core barrier for maintaining the health of the tooth structure and periodontal mucosa; an imbalance in the microbiome can induce dental caries and periodontal inflammation and, through mucosal immune pathways, contribute to systemic metabolic and immune disease processes (21–24). The extreme environmental conditions unique to the Qinghai–Tibet Plateau—including hypoxia, low temperatures, and intense radiation—exert strong selective pressures on oral microorganisms, significantly altering their diversity and community structure (25). However, previous studies have focused primarily on adults, and the oral microbiome characteristics of children aged 3–5 years—a critical window for microbial development—as well as the association between microbial imbalance and pediatric oral diseases remain unclear. In this study, stratified and grouped regression models adjusted for multiple confounding variables were used to investigate the associations of altitude and ethnicity on the oral microbiome of preschool children to identify characteristic oral microbiome phenotypes in children living at high altitudes and in lowland areas, and, in conjunction with the pathogenic mechanisms of pathogenic bacteria, to elucidate how these microbiome differences correspond to the risk of developing dental caries and periodontal inflammation.

Baseline data indicate that there were imbalances among the three groups of children in terms of daily sugar intake, dietary pattern, residential area, and maternal socioeconomic indicators. While these variables have been shown to alter the oral microbiome, after adjustment via multiple linear regression, none of the confounding factors independently influenced microbial diversity or the abundance of core species; only altitude had a highly significant effect on α-diversity and the abundance of multiple dominant species. These results suggest that after adjusting for confounding variables, altitude exhibits strong statistical associations with shifts in children's oral microbiota, with association magnitudes exceeding those of ethnic factors; These altitude-associated microbial differences may reflect variation in disease-related microbiota characteristics, ultimately altering children's susceptibility to oral diseases by reshaping the microbial community structure.

In this study, the α-diversity of the oral microbiota in children living at high altitudes was significantly greater than that in children living at low altitudes, which contrasts with previous reports of reduced oral microbiota diversity in adults living on plateaus (10, 11). We speculate that this “reverse” difference may stem from the unique developmental characteristics of the microbiota during childhood. In infants and toddlers aged 0–3 years, the microbiota is influenced primarily by feeding methods and dietary patterns (14, 26). The period from 3 to 5 years of age represents a critical window during which the oral microbiome transitions from early colonization to an adult-like steady state (27, 28). During this stage, the microbiome exhibits greater plasticity and environmental sensitivity (14, 29–31), and environmental stresses (such as low oxygen and low temperatures) may be linked to positive adaptive selection during this period. Moreover, children living at high altitudes often come from rural areas, where the complexity of the living environment is positively correlated with microbial diversity (32), which may also promote microbial colonization and maintenance. Notably, despite this divergent pattern in α-diversity, taxonomic profiles (enrichment of *Prevotella*, *Veillonella* at high altitude and *Streptococcus* at low altitude) are largely consistent with prior reports (10, 11). However, the higher α-diversity seen in our high-altitude preschoolers contradict observations in high-altitude adults. This discrepancy likely arises from age-dependent microbiome plasticity: the immature oral microbiome in 3-5-year-olds may gain richness under high-altitude exposure, whereas the homeostatic adult microbiome tends to show reduced diversity under chronic high-altitude stress.

The ASV-based Venn-diagram results further corroborated these richness-related observations. Substantially higher total ASV counts were detected in both high-altitude groups, consistent with their elevated alpha-diversity. Only a limited set of 846 core ASVs was shared across all three groups, indicating that most ASVs were either group-specific or shared between only two groups. Notably, the number of ASVs shared exclusively between the two high-altitude groups (HH and HT) far exceeded the number shared between the low-altitude LH group and each high-altitude group, suggesting that children living at high altitude tended to harbors more overlapping ASV features irrespective of ethnicity.

In contrast, children living at low altitudes exhibit reduced microbial diversity and dominant colonization by *Streptococcus* species, accompanied by predicted enrichment of glycolytic pathways. The dominance of a single cariogenic bacterium occupying an ecological niche, coupled with a lack of microbial diversity, disrupts the balance of the oral microbiome, further amplifying the cariogenic damage caused by sugar intake. This pattern may be associated with higher potential caries risk among children living in low-altitude regions. Furthermore, multiple regression analysis confirmed that ethnic factors have no independent effect on α-diversity, further suggesting that environmental stress may be more strongly associated with children's oral microbial diversity and community structure (33).

At the community-structure level, altitude was independently associated with greater Bacteroidetes abundance after FDR correction. Although Firmicutes showed a nominal negative association with altitude, this association did not remain significant after correction. The distribution of bacterial genera exhibits distinct altitudinal stratification: children at high altitudes are dominated by *Prevotella melaninogenica*, whereas Han Chinese children at low altitudes are dominated by caries-associated *Streptococcus* species (34). This difference may be related to adaptation to high-altitude environments; *Prevotella* is a typical facultative anaerobe that exhibits a growth advantage under hypoxic conditions (35). This bacterium has the potential to induce pro-inflammatory cytokine secretion via the NOD2/NF-κB axis (36), which is consistent with the predicted enrichment of NOD-like receptor signaling related gene sets among our high-altitude subjects. This points to a potential capacity of plateau-resident oral microbiota to drive mucosal immune responses; nevertheless, this inference relies solely on 16S-based functional prediction and awaits validation using multi-omics profiling and measurements of host immune biomarkers. High expression of protein digestion and absorption pathways may facilitate the breakdown of oral epithelial proteins by *Prevotella* for nutrient acquisition, further expanding the colonization of anaerobic pathogenic bacteria and creating a cycle of “hypoxic environment–anaerobic bacterial enrichment–chronic mucosal inflammation.” Ethnicity was not significantly associated with the tested species after FDR correction, and the present findings do not support an independent effect of ethnicity on periodontal susceptibility.

LEfSe analysis revealed that Tibetan children living at high altitudes have enrichment of two major periodontal pathogens: *Porphyromonas gingivalis* and *Fusobacterium periodonticum*. However, in the regression model, the independent effect of ethnicity on *Fusobacterium periodonticum* did not reach statistical significance, suggesting that this difference may be influenced by the synergistic effects of altitude and lifestyle, and altitude shows the strongest statistical association.

The functional prediction results fully explain the molecular basis for the differentiation in oral disease profiles between children at high and low altitudes: Han children at low altitudes exhibit an enrichment of *streptococci* and predicted enrichment of glycolytic pathway, with early childhood caries being the core disease risk. It should be emphasized that these inferences concerning disease risk are derived from whole-saliva microbiome profiles and do not constitute direct evidence for pathogenic communities localized within dental plaque or gingival crevices. Combining species contribution analysis with baseline data explains this phenomenon: Han Chinese children at low altitudes have higher daily sugar intake levels, resulting in a greater abundance of oral *Streptococcus* species. *Streptococci* rely on highly efficient glycolytic pathways for energy production (37) and gain an ecological competitive advantage in a high-sugar oral microenvironment, which leads to a greater risk of dental caries in preschool children. In children living at high altitudes, anaerobic bacteria are enriched, and proinflammatory immune pathways are predicted to be enriched, with the core disease risk being chronic inflammation of the gums and periodontium (38, 39). These findings may inform clinical considerations for oral-health prevention and control as follows: for children aged 3–5 years in lowland areas, oral interventions should focus on the control of sugars, the suppression of cariogenic *streptococci,* and supplementation with acid-neutralizing beneficial bacteria to reduce the incidence of dental caries; for children in high-altitude regions, interventions should focus on regulating the abundance of anaerobic bacteria, alleviating chronic mucosal inflammation, and conducting regular screening of the gingival mucosa to prevent the progression of periodontal damage. The taxonomic signatures uncovered in this study can serve as candidate targets for future microbiome focused intervention research. Subsequent *in vitro*, animal, and human studies are warranted to assess whether agents such as *Streptococcus* salivarius K12, arginine, lactoferrin, and short chain fatty acid modulators can modulate oral microbial profiles and oral health outcomes in children residing at different altitudes.

Notably, this study included children with no clinically active caries or obvious periodontal inflammation; therefore, the association between the microbiome and disease represents logical inferences and hypothesis generation based on pathogenic mechanisms rather than a direct prediction of disease outcomes. This study has certain limitations. First, as a cross-sectional observational study, it can confirm only the correlations between altitude, ethnicity, and the microbiome; it cannot establish a causal relationship. Second, since the study included healthy children without oral diseases and did not concurrently collect clinical indicators of caries or periodontal inflammation. moreover, unstimulated saliva samples were used, which cannot fully represent dental and subgingival biofilms. Thus, our findings reflect only salivary-level microbial trends, and quantitative evidence linking microbiome characteristics to disease severity is lacking. Third, the study lacked a low-altitude Tibetan control group, and therefore did not constitute a complete altitude-by-ethnicity factorial design, preventing complete separation of altitude- and ethnicity-associated contributions and formal evaluation of their interaction. Although the multivariable regression models adjusted for measured dietary, socioeconomic, and residential factors, they could account only for confounders that were measured and correctly specified. Residual confounding may remain because of measurement error in the questionnaire-based dietary data, unmeasured dietary characteristics such as the timing and frequency of intake, and other environmental, cultural, or lifestyle factors. Moreover, “ethnicity” in this study represents a composite of cultural, dietary, socioeconomic, environmental, and potentially host-related factors; therefore, the observed associations should not be interpreted specifically as genetic effects. These findings should accordingly be considered exploratory and require validation in a fully crossed study design. Fourth, 16S rRNA sequencing yields only taxonomic marker sequences; functional inference depends on homology mapping against reference databases and merely estimates the community's potential functional gene repertoire. By comparison, metagenomics directly sequences all microbial DNA to reveal genuine functional gene profiles. Accordingly, our observed KEGG pathway differences are taxon-based predictions, not actual sample gene contents.

Future research should (1) expand the cohort to include children with dental caries and gingival inflammation, collect clinical oral-disease metrics alongside local-niche samples such as dental plaque and gingival crevicular fluid, construct an association model linking “altitude–microbiome abundance–clinical indicators of oral disease,” and quantify the predictive power of the microbiome for disease onset; (2) include a low-altitude Tibetan control group to disentangle the main effects and interaction effects of altitude and ethnicity; (3) adopt metagenomic or multi-omics approaches to verify predicted functional pathways and capture real-world microbial gene content and metabolic activity; (4) conduct longitudinal follow-up to track the development of the microbiome from ages 3–and the incidence of new oral diseases in children aged 6–7, thereby validating the microbiome's long-term predictive role in disease; and (5) on the basis of the core cariogenic and proinflammatory bacterial species differing between high- and low-altitude regions, conduct further intervention studies to explore the potential of scenario specific oral microbiome modulating formulations to support precise pediatric oral health management.

## 5 Conclusion

This study systematically characterized salivary microbial community diversity, taxonomic composition, and predicted metabolic functions in 3–5-year-old Han and Tibetan children from plain and plateau settings. After adjusting for confounding variables, strong statistical associations were observed between altitude and preschool children's oral microbiome, with association magnitudes greater than those of ethnic factors. Children residing at high altitude exhibited higher oral microbial diversity together with enrichment of periodontal related anaerobes such as *Prevotella* and *Fusobacterium periodonticum* and predicted enrichment of NOD like receptor signaling pathways. Children living at low altitude showed enrichment of cariogenic *Streptococcus* alongside predicted enrichment of glycolysis driven acid producing pathways; distinct microbiome phenotypes were observed between the two groups. This study explores potential association-based framework linking “altitude environment–microbiome structure–risk of pediatric oral diseases,” providing a fundamental microbiological theoretical basis and identifying candidate pathogenic targets for stratified prevention and control of oral diseases and targeted microbiome interventions in preschool children across different altitude regions.

## Data Availability

Raw reads of the samples analyzed in this study have been deposited in the NCBI Sequence Read Archive (SRA) under BioProject accession number PRJNA1543217 (Submission ID: SUB16551936).
